# LAMAS (Light, Activity, Meals, & Sleep) timings & burnout, anxiety, and depression in teachers: Protocol for a cross-sectional study

**DOI:** 10.1371/journal.pone.0348121

**Published:** 2026-04-24

**Authors:** Marius König, Jonas P. Wallraff, Florian Glenewinkel, Ursula Wild, Thomas C. Erren, Philip Lewis

**Affiliations:** Institute and Policlinic for Occupational Medicine, Environmental Medicine and Prevention Research, Faculty of Medicine and University Hospital of Cologne, University of Cologne, Cologne, Germany; Public Library of Science, UNITED KINGDOM OF GREAT BRITAIN AND NORTHERN IRELAND

## Abstract

**Background:**

Teachers play a key role in society and make up ~1.5–2.5% of the working population. Yet, there is a teacher shortage in many countries and preventive occupational medicine strategies are called for. The primary objective of this project is to explore single and joint associations of the diurnal distributions of light, activity, meal, and sleep timing and work-related exposures with severity scores of burnout, anxiety, and depression in a cross-sectional study of secondary school teachers in Germany.

**Methods and analysis:**

The study will involve a one-time collection of questionnaire-based data on sleep, burnout, anxiety, and depression, sensor-based data on light and activity over one week, and diary-based data on work, sleep, and meals over one week. time. The protocol has been registered on the Open Science Framework (https://doi.org/10.17605/OSF.IO/U4R5M).

**Discussion:**

From a preventive occupational medicine perspective, identifying where and how light, activity, meal, and sleep timing may be targeted to mitigate burnout, anxiety, and depression could inform measures to be tested not only at the individual (micro) level, but also at systems (meso-institutions; macro-policy and society) levels.

## 1. Introduction

Teachers play a key role in society not only as educators, but also as mentors, role models, motivators, administrators, event organisers, guardians and care-givers, councillors, and social workers. They make up ~1.5–2.5% of the working population [1–4]. Therefore, keeping teachers healthy and happy is of paramount importance. Unfortunately, the demands of the job are harsh and, despite the high proportion of the working population being teachers, there is a teacher shortage in many countries [5]. A survey of European teachers found that more than half view their profession as unattractive [6]. Even more consider that the workload disallows a healthy work-life balance [6].

An impaired work-life balance prompts us to consider the diurnal distributions of lifestyle- and work- related exposures in teachers. By diurnal distributions of lifestyle-related exposures, we mean light, activity, meals, and sleep (LAMAS) and their timings. By diurnal distributions of work-related exposures, we mean times and durations of work-related activities. While there is no shortage of literature demonstrating high prevalence of high burnout, anxiety, and depression severity scores among teachers [7–9], there is scant information regarding their associations with diurnal distributions of LAMAS- and work-related exposures. This prompts us to consider burnout, anxiety, and depression severity scores as outcomes. LAMAS, in content, in volume, and in timing, can likely affect both somatic and mental health [10–14]. Delayed circadian timing is found in patients with mental health disorders and later chronotypes seem to suffer from psychological distress more frequently [15,16]. Light is a key time cue for circadian rhythms and sleep, sleep timing affects light exposures, and activity and meal timings may also act as time cues [13,14].

Preventive health promotion measures have been called for [5], but promotion puts agency on the individual. Meeting this agency is even more difficult when compounded by burnout, anxiety, and depression symptom severity. From a preventive occupational medicine perspective, identifying where and how LAMAS may be targeted to mitigate burnout, anxiety, and depression could inform measures not only at the individual (micro) level, but also at systems (meso-institutions; macro-policy and society) levels.

## 2. Objective

The objective of this project is to explore single and joint associations of the diurnal distributions of LAMAS- and work-related exposures with severity scores of burnout, anxiety, and depression in a cross-sectional study of secondary school teachers in Germany.

## 3. Methods & analyses

This is a cross-sectional study of secondary school teachers in the Cologne-Bonn region of the federal state of North-Rhine-Westphalia in Germany. The study will involve a one-time collection of questionnaire-based data on sleep, burnout, anxiety, and depression, sensor-based data on light and activity over one week, and diary-based data on work, sleep, and meals over one week. The cross-sectional design is justified for the following reasons: Our objective is exploratory, exposure tracking longitudinally presents practical constraints, and burnout (primary endpoint) is not a momentary state (i.e., burnout is a syndrome rather than a fleeting feeling that changes from one moment to the next) meaning that burnout severity score prevalence can be appropriately captured in a snapshot.

### 3.1. Ethics

This study will be reported in accordance with the Strengthening the Reporting of Observational Studies in Epidemiology (STROBE) statement [17]. Ethical approval has been granted by Ethics Commission of the Medical Faculty of the University of Cologne based on written informed consent and in accordance with the Declaration of Helsinki (Project Reference Number 24–1224). The participants were provided with an information document (approved by the above-mentioned Ethics Commission) and provided written informed consent after having read through the document and been given time to ask questions of the study coordinator. All individual data will be de-identified; only the principal investigator will have access to identifiable information. Participation is voluntary and participants are able to withdraw their consent at any time. The protocol has been registered on the Open Science Framework https://doi.org/10.17605/OSF.IO/U4R5M.

### 3.2. Recruitment

Secondary schools will be contacted and asked to participate in the study by non-probability (“convenience”) sampling. For secondary schools in this region, students typically enter at ~10 years old and compulsory education is until ~15 years old. Students who complete the lower secondary level may prepare for university-entry qualifications in the upper secondary level, finishing at ~19 years old. We do not include vocational schools (a form of upper secondary school geared toward preparing students to enter the workplace with specific skills). If a school’s representatives give positive feedback, all teachers will be invited to participate via the school’s internal general messaging service. With permission from the given school, appointments will be scheduled and data collected on site following participant informed consent.

To be as inclusive as possible, there are no restrictions beyond being currently employed and actively working as a secondary school teacher in the Cologne-Bonn region. We have no restrictions by part-time vs full time work nor by time-of-year of recruitment with the exception of school holidays. In other words, some teachers may participate in more or less busy/stressful times of year and the week of participation is not systematically chosen. We do not limit data collections to on site in schools – teachers can contact us independently with their interest to participate and we can schedule briefing and data collection for a non-school location. We anticipate that advertisement of our study will spread to other schools in our catchment area by word-of-mouth (friends who have studied together, professional development trainings, etc). We aim to include as many schools as possible in terms of different secondary levels within the German academic system, urban and rural, and different social index locations.

We are limited in terms of how many teachers can provide data at a given time due to the number of available light and activity sensors. We exclude primary and tertiary level educators to increase the homogeneity of our population. We expect primary and tertiary level educators to face different work requirements and workloads since they work with young children or adults, in comparison to secondary school teachers, who predominantly work with adolescents. Additionally, more than half of the teachers in Germany work in secondary education [4].

### 3.3. Data collection

**Fig 1:**
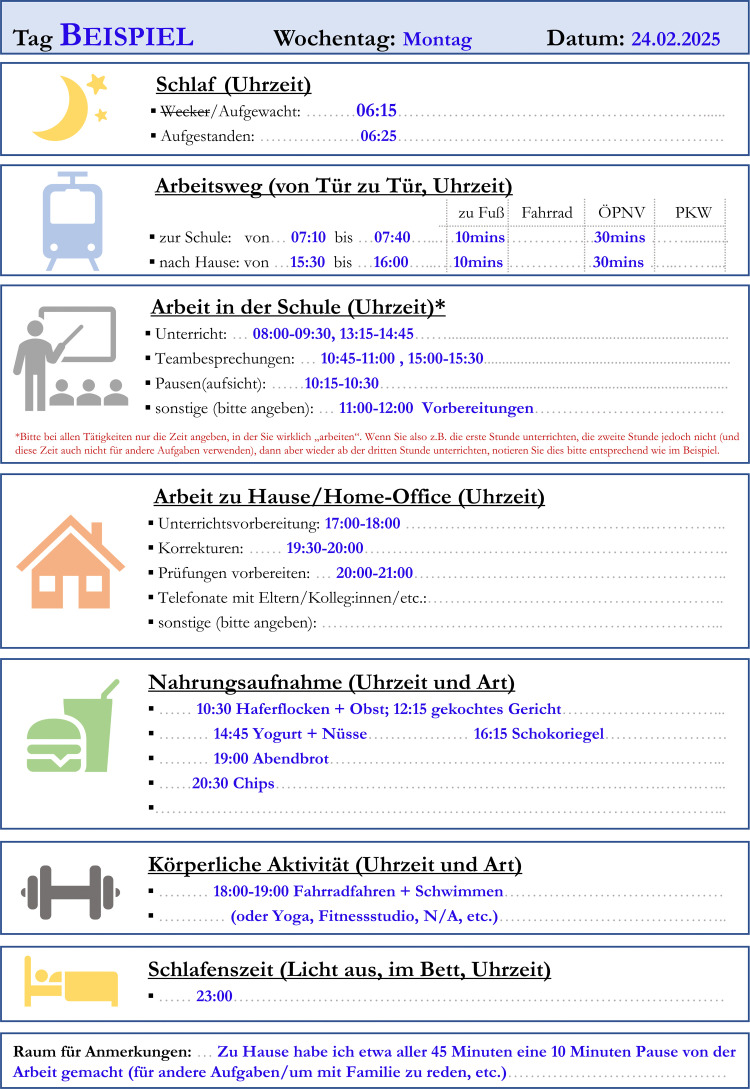
Example Diary Day. The diary entry is in German as it will be used in a German speaking population. Briefly, “Schlaf” = Sleep; “Aufgestanden” = Got out of bed; “Arbeitsweg” = Journey to Work; “Arbeit in der Schule” = Work in School; “Nahrungsaufnahme” = Nutrition Activities; “Körperliche Activität” = Physical Activity, “Schlafenszeit (Licht aus, im Bett)” = Sleep time (Lights out, in bed).

Teachers will be met individually or in small groups in a suitable (quiet) location (e.g., during free periods in schools). First the teachers will receive study information and will be given time to read it thoroughly and ask questions. If they provide consent to participate, they will be provided with questionnaires that take ~30 mins to complete. Following questionnaire completion, we will provide them with an activity and light sensor and a diary and instruct them on how to use them (Fig 1). After one week, we shall collect sensors and diaries.

### 3.4. Tools

All of the following questionnaires are validated for use in German-speaking populations and use simple continuous scale scoring methods. The Munich Chronotype Questionnaire (MCTQ) focuses on sleep timings on free days and work days to evaluate chronotype [18]. The Insomnia Severity Index (ISI) measures insomnia characteristics and symptoms, like subjective sleep disturbances, worries concerning disturbances, and impairments in daily life [19]. The Pittsburgh Sleep Quality Index (PSQI) evaluates sleep quality during the previous month, and is a broader construct than the insomnia severity evaluated in the ISI [20]. The Beck Depression Inventory (BDI) is used to assess the severity of depression symptoms [21]. The General Anxiety Disorder 7-Item scale (GAD-7) is used to assess the severity of anxiety symptoms [22]. The Maslach Burnout Inventory (MBI) is the most commonly used tool to assess burnout and its sub-components of emotional exhaustion, depersonalisation, and accomplishment.

A one-page questionnaire to assess demographic (e.g., age, sex, ethnicity) and work characteristics (e.g., subjects taught, years of experience, size of classes) will also be provided.

The provided diaries have sections for each of the 7 days to report the following: working times and task types, work locations, meal timings and contents, activity timings and contents, and sleep timing. An example for a completed diary entry is provided in Fig 1.

Light and activity data will be collected with use of the MotionWatch8 (CamNTech, UK). This is a medical grade, wrist-worn, combined light and activity sensor, similar in size, weight, and shape to a typical wristwatch. The activity component is a tri-axial accelerometer that provides an output as counts per selected time epoch (from 1–60 s). The light sensor provides an output of average lux per epoch, with a range of 0–64,000 lux. Participants will be instructed to wear the sensor continuously on their non-dominant wrist, just removing it in case of fully submerging the hand into water, e.g., when taking a shower or swimming. Data will be recorded in 30 s epochs, and the tri-axial mode 3 will be used for activity. The MotionWare software (Version 1.4.20, CamNTech, UK) will be used for data analyses.

### 3.5. Power analysis

Our power calculation estimates what effect size differences in emotional exhaustion severity (a key component of burnout) would be detectable for different sample population sizes between an evenly split assumed “exposed” group and an “unexposed” group (e.g., unexposed defined as ≤ median intensity exposure and exposed defined as > median exposure), if the unexposed group is assigned a mean population based emotional exhaustion score of 2.4 ± 1.3 (mean ± standard deviation) [23], a common standard deviation is used across evenly split groups, α = 0.05, and β = 0.2. Sample sizes of 100, 200 and 300 participants would yield detectable effect size differences of 0.74 (moderate-large), 0.52, and 0.42 (moderate-small), respectively. The control group will be an overestimate as it based on a mean population score [23]. A moderate effect size difference of 0.5 (corresponding to ~0.65 points of emotional exhaustion) would be relevant given the sizeable teacher population, and that a decrease of this size would mean shifting to lower severity categories for many. Thus, our aim is recruit ~200 participants. Mean and standard deviations of anxiety and depression scores have lower spreads, so even smaller effect size differences would be statistically detectable with ~200 participants.

### 3.6. Data analysis

Analyses will be performed with Stata/SE-17 (StataCorp LLC, USA). Descriptive statistics for demographics, light exposure, activity, and questionnaire scores/results will be generated. Comparisons of mean and medians of LAMAS variables and outcomes by demographic and work characteristics will be conducted. Associations between LAMAS variables and outcome variables (i.e., burnout, anxiety and depression severity scores) will be explored in multivariable regression models. Joint analyses will also be conducted.

Diurnal distributions of LAMAS variables associations with outcomes will be explored by several means. These will include: (i) assessing features of multi-component cosinor models with nested generalised linear model approaches and predictions at shifted diurnal profiles that are identified from k-means cluster analyses; (ii) assessing exposure and relative exposure volumes during school times, after school times, public holidays and weekends, and ratios between these time periods; (iii) assessing theoretical exposure volume diurnal redistributions to different times of day using quasi-compositional data (iso-temporal substitution) analyses. The latter can be achieved by partitioning mean total daily exposure into 4-hour time blocks of the 24-hour day (starting with, e.g., 04:00 or with time of awakening), expressing proportions as totals of exposure and using regression models that include mean total daily exposure and omitting one time block; thus, coefficients for each bin will represent the association with reallocation of exposure from the reference bin (rescaled predictors will be used to allow assessing percentage of reallocation rather than total time block reallocation). Theoretical dose responses will be assessed using increasing percentage reallocations. Sensitivity analyses will include using different time-block durations.

In terms of analytical prioritisation, our primary analysis involves the joint effects of the diurnal distributions of LAMAS (as primary exposures) on associations with burnout severity scores (as primary outcome). We expect that diurnal distributions of LAMAS and work exposures that lean toward higher intensities or proportions later in the day relative to earlier in the day (with the exception of sleep) will be associated with higher burnout symptom severities. As our analyses are exploratory, the analytical approaches are complementary, and we do not want for potentially meaningful patterns to be obscured, we shall not make adjustments for multiple tests.

Diary timing data will be checked against data from activity and light sensors. Misclassified diary timing data will be corrected in accordance with sensor data. Missing activity sensor data (e.g., such as removal for swimming that corresponds with swimming time indicated in the diary) will be imputed based on context (e.g., using plausible counts based on within-subject data from a similar physical activity). Missing physical activity data > 10 minutes and corresponding with no indicated activity will be deemed non-wear time. Unusual light exposures (e.g., low lux accompanying high physical activity from the sensor and diary indicated outdoor activity due to, for instance, covering of the sensor) will also be imputed based on context (e.g., appropriate lux levels for the given time of day and location). Results will be reported for sensor data with and without imputations.

Confounding and effect modification will be explored using age, sex, ethnicity, chronotype, subjects taught, school form, years of experience, class sizes, official working hours per week, presence of chronic disease, taking medication, smoking and alcohol consumption, recent jet-travel across time zones in the previous two weeks.

### 3.7. Timeline

Regarding recruitment, the first participant was recruited on 05 March 2025. Forty-four participants have been recruited to date. Recruitment is expected to continue through to 31 December 2026. This is based on the number of sensors we have at our disposable, the individual monitoring periods, assumes we can collect data from new participants every 14 days during the school terms and for all sensors to be in use, and allows a buffer for unexpected pauses or difficulty in recruiting for all sensors to be in use. No data has yet been analysed. Completed study results are expected in 2027.

### 3.8. Key assumptions of the study

This study assumes that the short-term measurements capture stable patterns sufficiently to detect meaningful associations without assuming directionality. Regarding population, a systematic non-response bias among the working teacher participants is not assumed. Accuracy of sensors and honest and accurate responses to questionnaires and in diary entries are assumed where necessary (i.e., checks will be made wherein sensor and self-reported data overlap). Context-dependent assumptions including that week-specific external events did not systematically bias results and that work demands were typical though time-of-year differences will be explored to this end if and where this is possible.

## 4. Discussion

There is an abundance of literature concerning burnout, anxiety, and depression in secondary school teachers, yet very little that focuses on feasible targets for preventive occupational medicine. This study addresses this gap by exploring LAMAS- and work-related exposures and their timings for associations with burnout, anxiety, and depression. Identifying where and how LAMAS may be targeted to mitigate burnout, anxiety, and depression could inform measures not only at the individual (micro) level, but also at systems (meso-institutions; macro-policy and society) levels. Of course, any such measures would require confirmation from longitudinal and/or experimental study designs.

Our expectations are that diurnal distributions of LAMAS and work exposures (from factual and theoretical analyses) that lean toward higher intensities or proportions earlier in the day relative to later in the day will be associated with lower symptom severities, with the exception of sleep.

The analyses plans are extensive, but using multiple analytical methods that target the same question may be considered as forms of sensitivity analyses. They are also part of the exploratory nature of this study; we note that there are many degrees of freedom, but all results will be reported. More targeted studies will be needed to assess validity of the findings.

The targeting of diurnal distributions of LAMAS and work-related exposures will be translatable to many other occupations for whom home-office hours and ‘constant on’ due to increased digitalisation are becoming increasingly the norm. The data and results may also be of interest to the fields chronobiology and chrono-epidemiology.

Overall, this study – with added translational value for other fields and occupations – will inform how to develop and test in longitudinal and/or experimental study designs potential preventive occupational medicine strategies that target LAMAS- and work-related exposures to help secondary school teachers against burnout, anxiety, and depression.
